# Neuroplasticity in F16 fighter jet pilots

**DOI:** 10.3389/fphys.2023.1082166

**Published:** 2023-02-15

**Authors:** Wilhelmina E. Radstake, Steven Jillings, Steven Laureys, Athena Demertzi, Stefan Sunaert, Angelique Van Ombergen, Floris L. Wuyts

**Affiliations:** ^1^ Radiobiology Unit, Belgian Nuclear Research Centre SCK CEN, Mol, Belgium; ^2^ Laboratory for Equilibrium Investigations and Aerospace, University of Antwerp, Antwerp, Belgium; ^3^ Coma Science Group, GIGA Consciousness, GIGA Institute, University and University Hospital of Liège, Liège, Belgium; ^4^ Physiology of Cognition Lab, GIGA-CRC In Vivo Imaging, University of Liège, Liège, Belgium; ^5^ Psychology & Neuroscience of Cognition, University of Liège, Liège, Belgium; ^6^ Translational MRI, Department of Imaging and Pathology, KU Leuven and University Hospital of Leuven, Leuven, Belgium; ^7^ Department of Translational Neurosciences—ENT, University of Antwerp, Antwerp, Belgium

**Keywords:** resting state fMRI, fighter pilots, neuroplasticity, gravity transitions, brain, MRI

## Abstract

Exposure to altered g-levels causes unusual sensorimotor demands that must be dealt with by the brain. This study aimed to investigate whether fighter pilots, who are exposed to frequent g-level transitions and high g-levels, show differential functional characteristics compared to matched controls, indicative of neuroplasticity. We acquired resting-state functional magnetic resonance imaging data to assess brain functional connectivity (FC) changes with increasing flight experience in pilots and to assess differences in FC between pilots and controls. We performed whole-brain exploratory and region-of-interest (ROI) analyses, with the right parietal operculum 2 (OP2) and the right angular gyrus (AG) as ROIs. Our results show positive correlations with flight experience in the left inferior and right middle frontal gyri, and in the right temporal pole. Negative correlations were observed in primary sensorimotor regions. We found decreased whole-brain functional connectivity of the left inferior frontal gyrus in fighter pilots compared to controls and this cluster showed decreased functional connectivity with the medial superior frontal gyrus. Functional connectivity increased between the right parietal operculum 2 and the left visual cortex, and between the right and left angular gyrus in pilots compared to controls. These findings suggest altered motor, vestibular, and multisensory processing in the brains of fighter pilots, possibly reflecting coping strategies to altered sensorimotor demands during flight. Altered functional connectivity in frontal areas may reflect adaptive cognitive strategies to cope with challenging conditions during flight. These findings provide novel insights into brain functional characteristics of fighter pilots, which may be of interest to humans traveling to space.

## Introduction

To be able to functionally adapt to changing environments, the brain is capable of altering its function and structure. This process is called neuroplasticity and is crucial for skill learning and adaptation (Pearson-Fuhrhop and Cramer, 2010). How these adaptations occur under changing gravitational conditions is of increasing concern with plans for future space missions to the Moon, Mars and eventually beyond. Linear accelerations, including gravity, are detected by the otolith organs, which together with the three semicircular canals (SCC) form the vestibular system. The otoliths are stimulated by the gravito-inertial acceleration (GIA), which is the sum of all linear accelerations, including the gravitational acceleration, and serves as a vertical reference axis. The SCCs are sensitive to angular accelerations, enabling the detection of head rotations. The vestibular system therefore plays a prominent role in adapting to altered gravitational environments, though also its close interplay with the visual and proprioceptive senses for spatial perception functions are important in mediating adequate adaptations.

Pilots offer a nice model on Earth to investigate how challenging gravitational environments lead to sensory adaptations. During in-flight coordinated turns (banking maneuvers), fighter pilots undergo a body tilt in the roll plane and simultaneously experience centrifugal forces (Demir and Aydın, 2021). In this case, the GIA is the vector sum of the centrifugal force during the maneuver and the gravity force. The GIA is therefore tilted with respect to Earth’s gravity vector, though the pilot is tilted in the same plane and direction. Consequently, the tilted position of the otoliths and the tilted GIA remain matched and the pilot will not experience the roll tilt based on otolith signaling only. On the contrary, the SCCs are stimulated by angular accelerations, and therefore they do signal the pilot’s tilt in the roll plane during the maneuver. This causes an intravestibular conflict during the banking maneuvers, triggering adaptation in fighter pilots. Previous studies demonstrated that fighter pilots have increased sensitivity to tilts in the pitch and roll plane compared to matched controls (Tribukait et al., 2011; Tribukait and Eiken, 2012). Also in the case of microgravity, the vestibular system needs to adapt because the otoliths are no longer stimulated by head tilts due to the absence of a perceived gravity force during spaceflight, while the SCCs do respond to the angular acceleration.

At the central nervous system level, vestibular information is integrated with visual and proprioceptive information to render a spatial perception of the body within the environment. This internal representation is constantly updated by sensory input, and it is also the basis for expected body positions. When a mismatch arises between the expected percept and the updated signals, spatial disorientation occurs and often also motion sickness. This interplay is also challenged during flight. For example, when flying in a homogeneous visual scene that does not contain orientational reference cues, various maneuvers and gravitational alterations can induce spatial illusions in pilots (Demir and Aydın, 2021), and spatial disorientation is a known cause of fatal accidents of pilots (Gibb et al., 2011). During spaceflight, unloading of the body and a mismatch between expected and actual multisensory feedback from body movements also give rise to conflict signals in the brain. The brain will then adapt to the intersensory conflicts by altering the processing of sensory input (Kornilova and Kozlovskaia, 2003). Yet, these phenomena appear to decrease throughout time with training, after several days in microgravity, and by repeated exposure to microgravity, providing evidence for vestibular adaptations (Reschke et al., 1998; Schoenmaekers et al., 2022). Several hypotheses have been put forward to explain the origin of space motion sickness. One is the otolith organ tilt-translation reinterpretation, which states that the sensory conflicts causing motion sickness triggers the brain to interpret otolith signals as translations only (Young et al., 1984). Alternatively, otolith signals that provide information on head position may be completely disregarded to avoid sensory conflicts (Guedry et al., 1998). Another theory states that the altered vestibulo-ocular reflex gains cause visual-vestibular conflicts in microgravity because of the discrepancy between expected and observed inputs.

Several neuroimaging studies have provided evidence of cortical adaptation to experiencing altered gravity levels in various settings. One study reported functional connectivity changes in sensorimotor, visual, proprioceptive, and vestibular regions when administering somatosensory stimulation to the foot soles during functional MRI scanning. These changes were interpreted as reorganization and reweighting of multisensory systems (Pechenkova et al., 2019). Another study used a skull tapping technique during functional MRI scanning, which stimulates the peripheral vestibular system. They reported post-flight changes in connectivity in sensorimotor, frontal, and occipital regions, further supporting the notion of multisensory compensation and reweighting in response to intersensory conflict (Hupfeld et al., 2022). Another study found that the right angular gyrus, a brain region involved in multisensory integration and verticality perception, exhibits decreased functional connectivity with the rest of the brain after first-time exposure to gravitational transitions induced by parabolic flight (Van Ombergen et al., 2017a). Finally, one study investigated neural correlates of fighter pilots, though with a specific focus on expert cognitive control. The authors showed that the medial superior frontal gyrus showed white matter structural changes that correlated with cognitive performance in fighter pilots (Roberts et al., 2010). These few studies indicate the occurrence of cortical functional adaptation to various challenging conditions that include gravity level changes.

How the brain adapts to overcome the various sensory and sensorimotor challenges during (space) flight requires further research, which is restricted due to the rarity of populations that can be tested in this setting, such as space crew, parabolic flight participants, and fighter pilots. This study aims to contribute to this line of research and investigates whether fighter pilots show altered functional connectivity (FC). First, we aimed to characterize which brain regions show FC changes that are associated with increasing experience in flying a fighter jet. Next, we sought to characterize FC characteristic differences between pilots and non-flying matched controls through an exploratory approach on the one hand, and through a hypothesis-driven approach on the other hand. For the latter approach, we specifically investigated whether a key vestibular hub in the cerebral cortex, the right parietal operculum (rOP2), showed connectivity differences between pilots and matched controls, based on the hypothesis of cortical vestibular adaptation due to gravity alterations. We also tested for connectivity differences between pilots and controls in the right angular gyrus (AG), based on the altered connectivity that was observed in this region after parabolic flight and based on its hypothesized role in adapting to altered gravity levels at a multisensory integration level.

## Methods

### Subjects

Fighter jet pilots were recruited *via* the Belgian Air Force. Inclusion criteria were age between 18 and 65. Exclusion criteria were: neurological disease, medication with effects on the CNS, excessive alcohol- and/or drug use, vestibular problems, jetlag (at least 1 week after transcontinental flight/mission) and at least 24 h since last exposure to high g-levels. A total of 10 male fighter pilots were included (mean age (SD) = 29 (3.2) years, range 23–32 years), who had on average 1,025 h of flight experience in an F16 fighter jet (SD = 595; range from 200 to 2,116 h). A control group (mean age (SD) = 29 (3.2) years, range 23–32 years) of 10 adults with no experience in flying was included, matched for age, gender, and educational level. Additionally, controls were also matched for handedness (9 right- and 1 left-handed in each group). All participants signed an informed consent form. The study was approved by the local ethics committee of the Antwerp University Hospital (13/38/357).

### Procedure

Prior to the scan session, participants completed the Edinburgh Handedness Inventory (Oldfield, 1971). A laterality index is calculated, where a score of 100 reflects complete right-handedness and a score of −100 reflects complete left-handedness. All participants completed an MRI safety screening form. For the resting-state functional MRI (rs-fMRI) acquisition, subjects were told to lay with their eyes closed, not to sleep, and not to think about anything in particular. Instructions were given prior to the experiment and at the start of the sequence. The pilots were asked prior to the experiment day to provide the number of hours flown in a F16 jet.

### Data acquisition

All data were acquired on a 3T Siemens MAGNETOM PrismaFit scanner (Siemens, Erlangen, Germany) located in the University Hospital of Antwerp. A 32-channel head coil was used for the acquisition of resting state data and T1 weighted images for anatomical reference.

The T1-weighted anatomical images were acquired using an MPRAGE sequence (TR = 2000 m; TE = 3.05 m; flip angle = 8°; voxel size = 1.0 × 1.0 × 1.0 mm). For rs-fMRI, 350 whole brain T2*-weighted images were acquired using a gradient-echo echo planar imaging sequence (TR = 748 m; TE = 31 m; flip angle = 70°; voxel size = 2 × 2 × 2mm; field of view = 212 × 212 × 144mm; matrix size = 106 × 106; number of slices = 72). rs-fMRI acquisition started with 4 dummy scans, which were immediately discarded to account for T1 saturation effects.

### Data preprocessing

Statistical Parametric Mapping 12 (SPM12; http://www.fil.ion.ucl.ac.uk/spm/) implemented in MATLAB R2019a (The Mathworks Inc., Natick, MA, United States) was used for data preprocessing for each participant. Functional and structural images were manually reoriented to assure rough spatial correspondence. Functional images were then realigned to the first volume, co-registered to the structural T1-weighted MRI, normalized into standard stereotactic Montreal Neurological Institute (MNI) space, and spatially smoothed using a Gaussian kernel of 6 × 6 × 6 mm^3^ full-width at half-maximum. Structural T1-weighted MRI data were segmented into gray matter (GM), white matter (WM), and cerebrospinal fluid (CSF) maps, which were non-linearly warped and normalized into MNI space.

For the resting state statistical analysis, the CONN v.19c functional connectivity toolbox (www.nitrc.org/projects/conn) was used. In addition to the preprocessing steps, rs-fMRI data were denoised. Motion parameters obtained during the realignment step, the motion outlier scans obtained from the artifact detection toolbox (ART; https://www.nitrc.org/projects/artifact_detect/), and their first temporal derivatives were used for denoising by entering them as regressors of no interest in the design matrix. 19 outlier scans in the control- and 13 in the pilot group related to motion were detected and removed from the data. Bandpass filtering of 0.008–0.09 Hz was applied, as well as linear detrending. The anatomical component-based noise correction (aCompCor) was used for further noise reduction. This method models the influence of noise as a voxel-specific linear combination of multiple empirically estimated noise sources by deriving principal components from noise regions of interest and including them as nuisance parameters within the first-level general linear model. Signal fluctuations arising from non-neuronal activity in the WM and CSF masks were included as noise ROIs (Behzadi et al., 2007).

### First-level analysis

For each subject, the Intrinsic Connectivity Contrast (ICC) was computed for each voxel in the brain (Martuzzi et al., 2011). This computation is based on a voxel-to-voxel correlation matrix, where correlation coefficients were first normalized to z-values in order to obtain a Gaussian distribution. The ICC of a voxel is then calculated as the average squared z-value of that voxel with all other voxels in the brain. As such, we obtained a whole-brain ICC map, where the voxel intensity reflects the degree to which that voxel is connected with the rest of the brain (Martuzzi et al., 2011).

Next, a seed-to-voxel or region-of-interest (ROI) analysis was adopted to investigate functional connectivity (FC) changes between a seed ROI and the rest of the brain. We chose as ROIs the resulting clusters from the ICC analysis, the main vestibular cortical area in the rOP2 (Eulenburg and Eickhoff, 2012) obtained from the archive of neuroimaging meta-analyses (http://anima.fz-juelich.de), and the right AG obtained from a previous study using parabolic flights (Van Ombergen et al., 2017b). The number of ROIs was restricted to these specific and well-defined areas to prevent accumulation of multiple comparisons. ROI-to-voxel correlation coefficients were Fischer transformed into z-values.

### Second-level statistical analyses

First, we performed a voxel-level correlation analysis between the number of hours flown in a fighter jet and the ICC measure. Next, we performed two-tailed unpaired two-sample t-tests between F16 pilots and controls for the ICC analysis, as well as the ROI analyses, to investigate statistically significant differences between both groups. Age was included as a nuisance regressor. An uncorrected whole-brain threshold of *p* < 0.005 followed by a cluster-wise threshold of *p* < 0.05 corrected for multiple comparisons with family-wise error (FWE) was applied on the statistical parametric maps. Finally, to investigate the associated networks of the different ROIs, we performed a two-tailed one-sample *t*-test using all subjects of both groups. A whole-brain voxel-level threshold of *p* < 0.001 uncorrected was applied on the statistical parametric maps, with a cluster-level threshold of *p* < 0.05 corrected with FWE.

## Results

### Functional connectivity changes linked to flight experience

**FIGURE 1:**
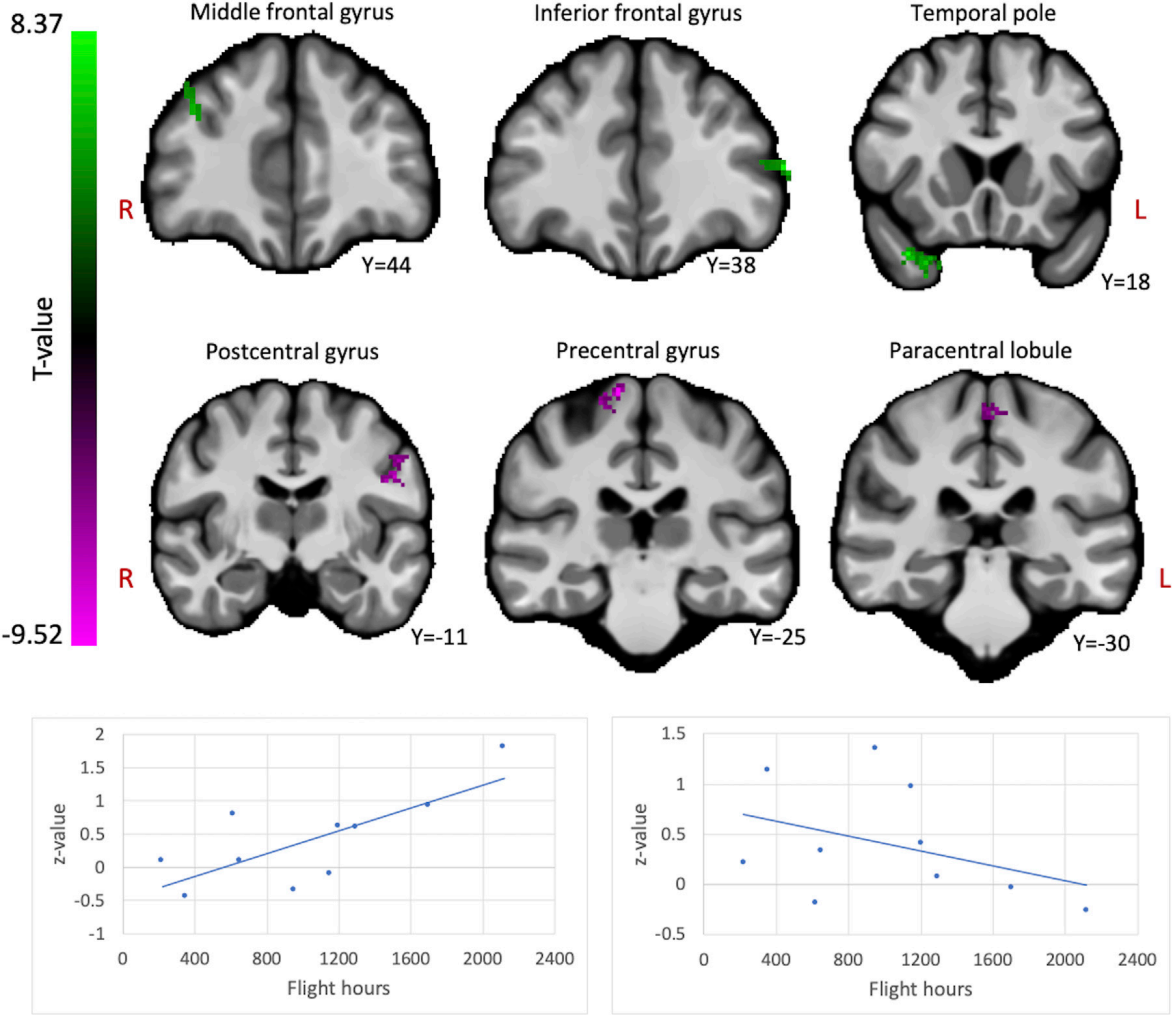
The brain shows positive and negative correlations between global connectivity and cumulative flight hours. Positive correlations between ICC values and flight hours are illustrated in green, whereas negative correlations are illustrated in magenta. A threshold of *p* < 0.005 uncorrected at the voxel-level and *p* < 0.05 family-wise error rate at the cluster-level were applied. Age was regressed out of the analysis. Plots illustrate the average ICC values of all clusters showing positive correlations (left) and negative correlations (right) with flight hours.

We assessed whether the total amount of flight time, as a proxy for cumulative exposure to gravity transitions, is correlated with brain FC in fighter pilots. These analyses revealed a positive correlation between flight hours and the ICC values in the right middle frontal gyrus, left inferior frontal gyrus pars triangularis, and right temporal pole (Figure 1). These results indicate that these regions exhibit stronger connectivity with the rest of the brain in pilots who have flown more often. On the other hand, negative correlations were found between flight hours and bilateral pre- and postcentral gyri, as well as the paracentral lobule, indicating that these regions show less global connectivity in more experienced fighter pilots (Figure 1). Information on the significant clusters from this analysis can be found in Table 1.

**TABLE 1:** Cluster information of all performed analyses.

Brain region	BA	Peak voxel coordinates			Cluster size	T-value	p-cFWE
		x	y	z			
Intrinsic connectivity contrast (ICC): Pilots > Controls							
No significant results							
Intrinsic connectivity contrast (ICC): Controls > Pilots							
Inferior frontal gyrus, L (pars orbitalis)	BA47	−42	40	−10	68	5.80	0.031
Seed: inferior frontal gyrus, L: Pilots > Controls							
No significant results							
Seed: inferior frontal gyrus, L: Controls > Pilots							
Superior medial gyrus, L	BA9	0	46	46	222	4.89	0.001
Seed: right parietal operculum 2: Pilots > Controls							
Visual cortex (hOc3; V3), L	BA18	−32	−100	−04	143	7.31	0.014
Seed: right parietal operculum 2: Controls > Pilots							
No significant results							
Seed: right angular gyrus: Pilots > Controls							
Angular gyrus, L	BA39	−40	−66	36	309	4.74	<0.001
Seed: right angular gyrus: Controls > Pilots							
No significant results							
positive correlation between ICC maps and flight hours							
Temporal pole, R	BA38	34	16	−30	67	11.67	0.001
Middle frontal gyrus, R	BA9	38	48	36	57	7.17	0.005
Inferior frontal gyrus, L (pars triangularis)	BA46	−54	40	6	41	8.76	0.050
negative correlation between ICC maps and flight hours							
Postcentral gyrus, L	BA4	−50	−10	24	153	11.30	<0.001
Paracentral lobule, L	BA1	−8	−30	60	95	9.73	<0.001
Precentral gyrus, R	BA6	14	−24	72	65	8.22	0.002

Significant clusters for the intrinsic connectivity contrast analysis, the seed-to-voxel analysis, and the correlation analysis with flight hours are described by the brain region label, peak voxel coordinates in MNI, space, cluster size and statistical information; BA, brodmann area; T-values are cluster-averaged; p-cFWE, is the *p*-value after cluster-wise family-wise error (FWE) correction. R = right, L = left.

### Group differences in functional connectivity

**FIGURE 2:**
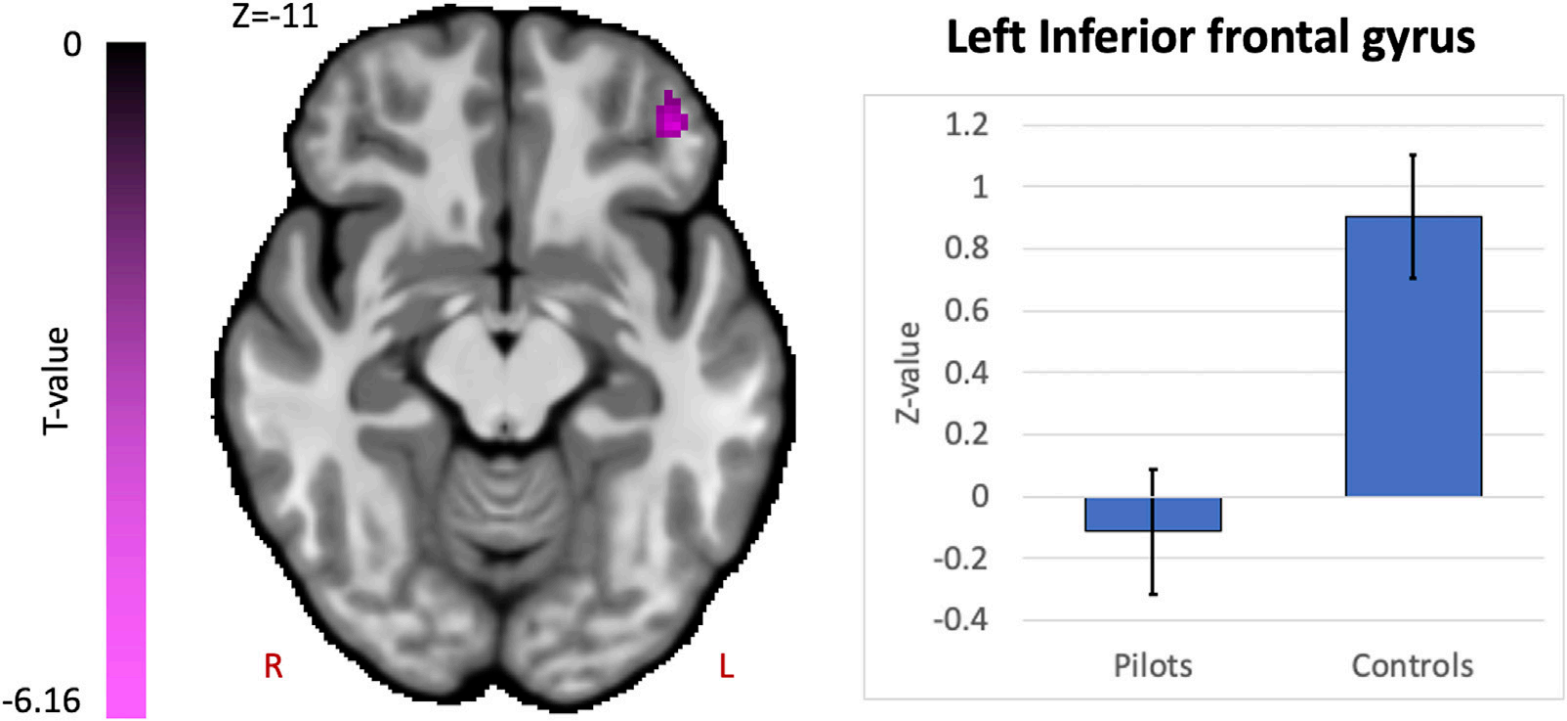
Hypothesis-free differences in global functional connectivity in fighter pilots compared to controls. The left inferior frontal gyrus (IFG) in fighter pilots showed decreased participation in whole-brain connectivity compared to controls, probed by the intrinsic connectivity contrast (ICC). Decreases in ICC are shown in magenta and are scaled by t-statistic (*p* < 0.005 uncorrected at the voxel-level, *p* < 0.05 family-wise error rate corrected at the cluster-level). The plot illustrates average effect sizes in the left IFG within each group (error bars indicate 95% confidence intervals). R = right, L = left.

**FIGURE 3:**
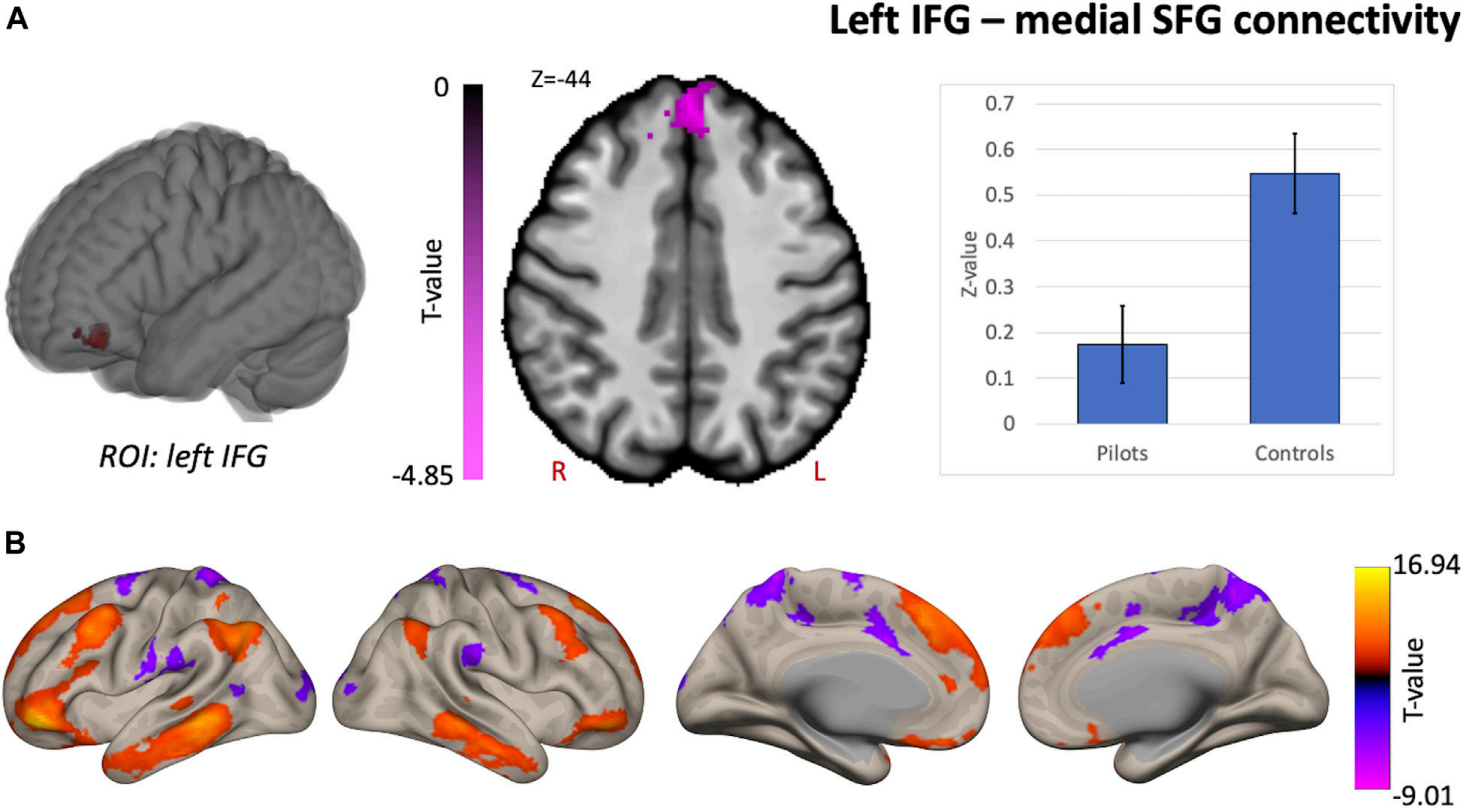
Supplementary analysis for comprehending the hypothesis-free results indicate that pilots and controls exhibit differential network-based organization. Post-hoc FC analysis with the resulting cluster from the intrinsic connectivity contrast (ICC) analysis as seed region. **(A)** Results from the comparison of pilots *versus* controls. Significant decreases are shown in magenta and are scaled by t-value (*p* < 0.005 uncorrected at the voxel-level, *p* < 0.05 family-wise error corrected at the cluster-level). The plot illustrates the average effect size of the FC between left inferior frontal gyrus (IFG) and the medial superior frontal gyrus (SFG) for pilots and controls (error bars indicate 95% confidence intervals). **(B)** Associated network of the left IFG illustrated by the positive and negative correlations with the rest of the brain in red-yellow and blue-purple respectively (*p* < 0.001 uncorrected at the voxel-level, *p* < 0.05 family-wise error corrected at the cluster-level). Results are scaled by t-value. R = right, L = left.

Comparing fighter pilots to controls, we found reduced global brain connectivity in the left inferior frontal gyrus (IFG) pars orbitalis, probed by the ICC metric (Figure 2). Post-hoc ROI analysis with the left IFG cluster as seed region revealed decreased FC with bilateral medial superior frontal gyrus (SFG) in pilots compared to controls (Figure 3A). Furthermore, we found that the left IFG was part of the frontoparietal network as described by Vincent and others (Vincent et al., 2008), with predominantly functional connections with dorsolateral prefrontal cortex, dorsomedial frontal cortex and inferior parietal lobule (Figure 3B).

**FIGURE 4:**
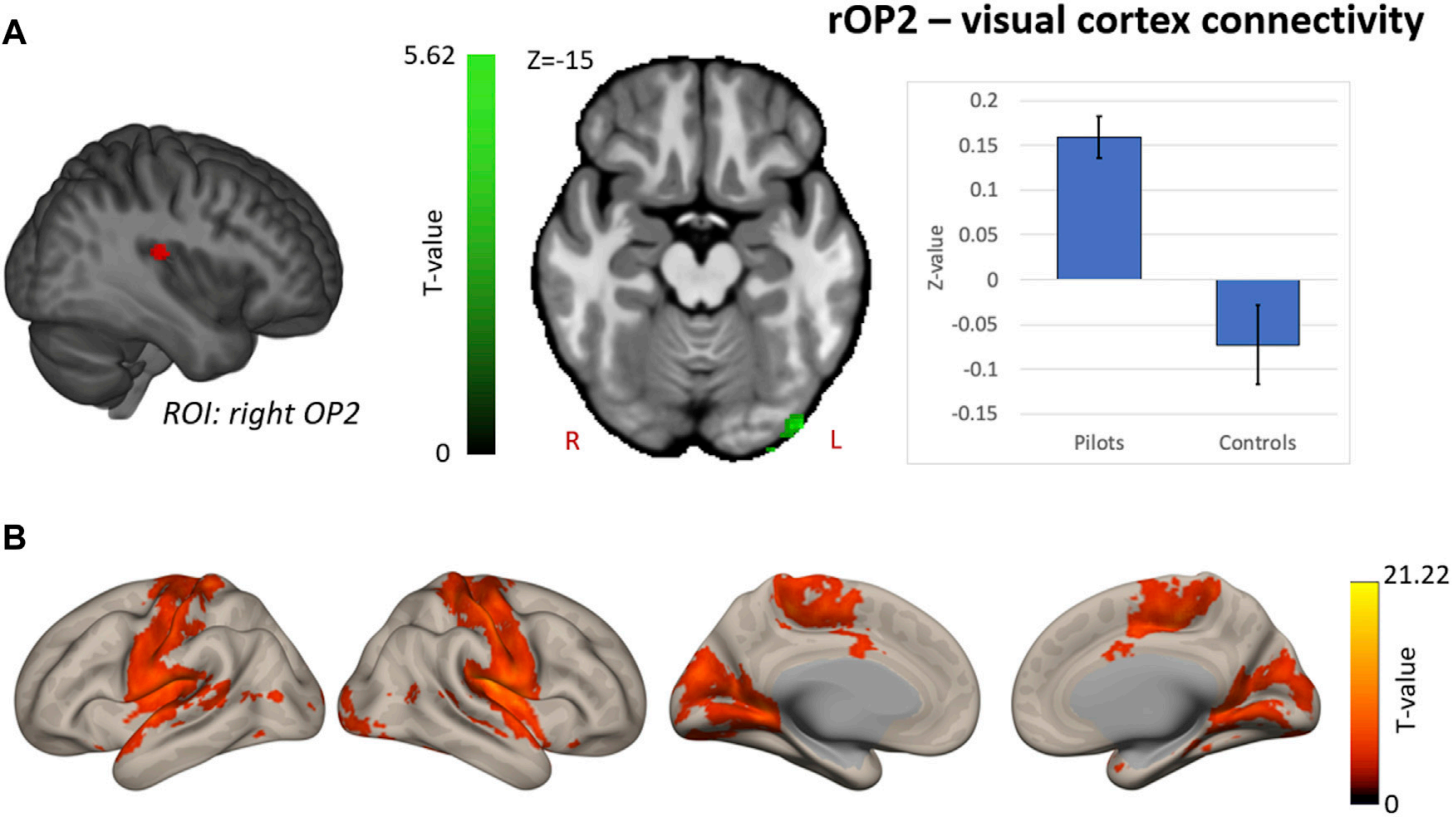
Hypothesis-driven analysis: Comparison of functional connectivity (FC) maps of the right parietal operculum 2 (OP2) between fighter pilots and controls **(A)** Pilots show greater FC between the right OP2 and the visual cortex. FC increases are shown in green and are scaled by t-value (*p* < 0.005 uncorrected at the voxel-level, *p* < 0.05 family-wise error corrected at the cluster-level). The plot illustrates the average FC values between right OP2 and the visual cortex for pilots and controls (error bars indicate 95% confidence interval). **(B)** The associated connectivity network of the right OP2 is shown as positive correlations with the rest of the brain in red-yellow (*p* < 0.001 uncorrected at the voxel-level, *p* < 0.05 family-wise error corrected at the cluster-level). Results are scaled by t-value. R = right, L = left.

**FIGURE 5:**
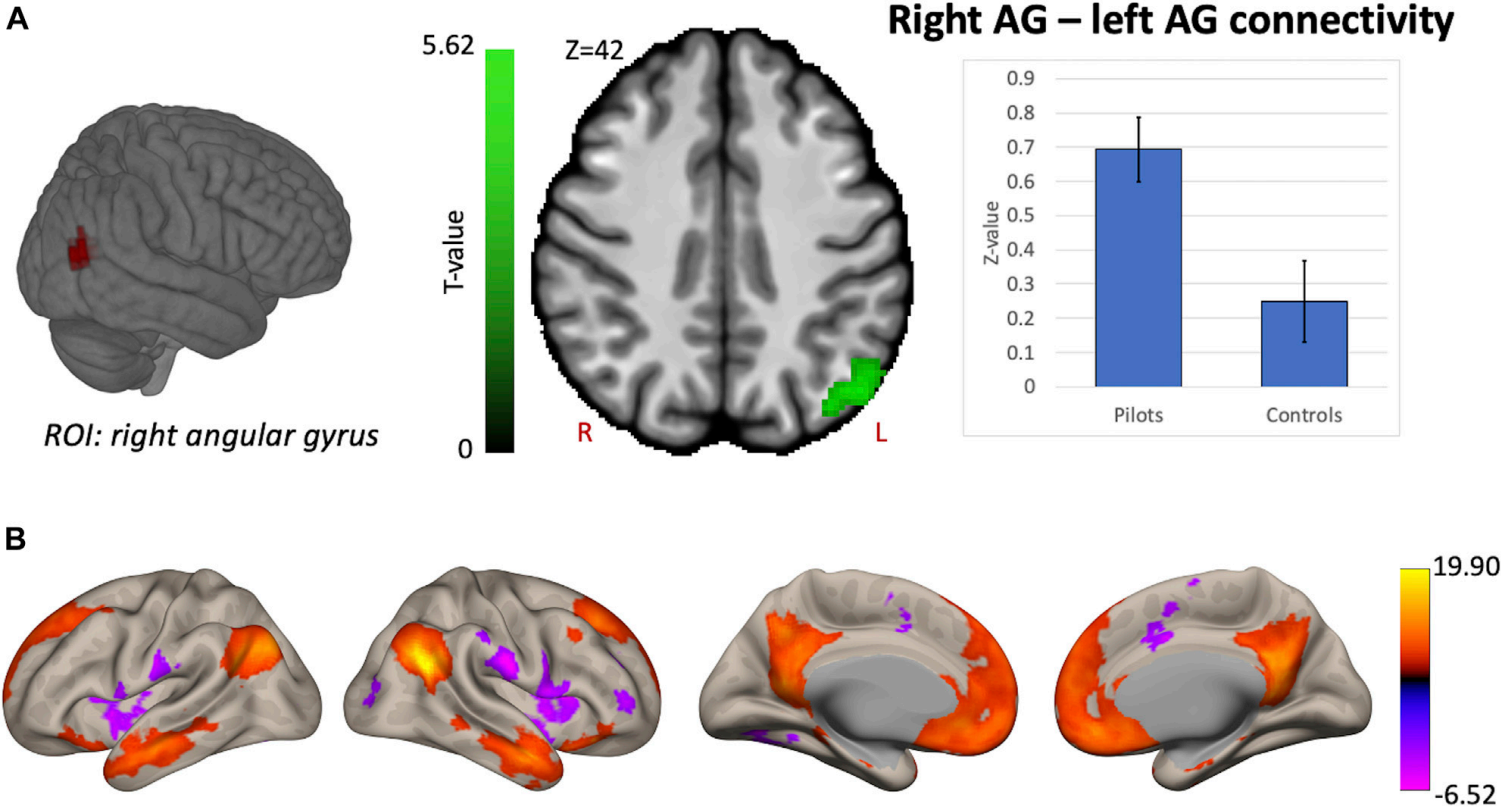
Hypothesis-driven analysis: Comparison of functional connectivity (FC) maps of the right angular gyrus (AG) between fighter pilots and controls. **(A)** Pilots show greater FC between the right AG and the left AG compared to controls. FC increases are shown in green and are scaled by t-value (*p* < 0.005 uncorrected at the voxel-level, *p* < 0.05 family-wise error corrected at the cluster-level). The plot illustrates the average FC values between right AG and left AG for pilots and controls (error bars indicate 95% confidence intervals). **(B)** The associated connectivity network of the right AG is shown as positive and negative correlations with the rest of the brain in red-yellow and blue-purple, respectively (*p* < 0.001 uncorrected at the voxel-level, *p* < 0.05 family-wise error corrected at the cluster-level). These results are scaled by t-value. R = right, L = left.

Hypothesis-driven ROI-based analyses were performed with the right OP2 and right AG as seed regions. We found increased FC between right OP2 and visual cortex in fighter pilots compared to controls (Figure 4A). The right OP2 showed statistically significant positive correlations with the bilateral pre- and postcentral gyri, visual cortex, and superior temporal gyrus (Figure 4B). Next, we found increased FC between the right AG and the left AG in pilots compared to controls (Figure 5A). The right AG shows predominant positive correlations with the left AG, posterior cingulate cortex, and medial prefrontal cortex, while it shows negative correlations with the anterior insular cortex (Figure 5B). These findings correspond with the right AG belonging to the default mode network. Information on the significant clusters from this analysis can be found in Table 1.

## Discussion

This study aimed to detect brain functional connectivity differences in fighter pilots compared to matched controls to provide insights into neuroplasticity due to exposure to altered gravity levels. This information is relevant for future long-duration missions, e.g. to the Moon with 0.16 g or to Mars with 0.38 g, where space crew will be exposed to varying levels of gravitational force across the span of the mission.

### Altered functional connectivity in more experienced pilots

First, we investigated which brain regions show altered global functional connectivity in proportion with the number of hours flown in a fighter jet. We found a negative correlation between the number of flight hours and connectivity of three regions in the sensorimotor gyri (precentral, postcentral, and paracentral gyri), suggesting these regions are less connected to the rest of the brain in more experienced pilots. Motor responses in altered g-levels has been investigated through a number of studies, demonstrating that study participants exert increased isometric responses (Sand et al., 2003) and perform less stable on flight simulator exercises (Guardiera et al., 2010) when being centrifuged at or above 3G. Considering fighter pilots, it was found that pilots also produced increased isometric responses in 3G, though this increase was less than in non-pilots (Guardiera et al., 2007; Guardiera et al., 2010). Additionally, novice pilots showed decreased flight stability during the first minute of 3G exposure, while experienced pilots did not (Dalecki et al., 2010). To better comprehend how exposure to increased g-force while operating a fighter jet could lead to the observed functional changes, an interesting follow-up to our current study could attempt to correlate neuroimaging findings with isometric motor responses and flight simulator performance during 3G centrifugation.

We also found positive correlations between number of flight hours and global connectivity in the right temporal pole, left inferior frontal gyrus, and right middle frontal gyrus. The middle frontal gyrus has a key role in executive cognitive control, working memory, attentional control, and conflict resolution (Owen et al., 1998; Milham et al., 2003; Wang et al., 2010). While the inferior frontal gyrus (pars triangularis) and temporal pole are often found to be implicated in semantic knowledge (Pobric et al., 2007; Pelletier et al., 2017; Chang et al., 2018), they are also both involved in conceptual knowledge (Baumgaertner et al., 2007; Ralph et al., 2009) and social and emotional processing (Olson et al., 2007; Park et al., 2010). Findings of increased global connectivity in these frontal regions in more experienced pilots might reflect the cognitive demands to overcome the challenges of operating a F16 fighter jet plane. However, an important remark here is that these regions all have many roles in human behavior and that it is beyond the ability of this study to ascribe one specific explanation for the observed correlations. Rather, the aim here is to characterize the brain regions that show modulated connectivity with increasing experience as a fighter pilot, with future studies being required to further elucidate the exact mechanisms.

### Functional connectivity alterations in prefrontal brain areas—Cognitive processing

Next, we found decreased global functional connectivity in the left IFG in fighter pilots based on a hypothesis-free exploratory approach, meaning that in pilots this region is less connected to the rest of the brain. Note that this IFG region does not overlap with the IFG cluster found to be positively correlated with flight hours, which otherwise would have raised the expectation of higher ICC values in pilots compared to controls, rather than lower values. Post-hoc ROI analysis reveals that this region is specifically less connected to the medial superior frontal gyrus (SFG) encompassing both hemispheres and that the left IFG belongs to a functional network resembling the frontoparietal control network (Vincent et al., 2008). This network is activated during controlled processing of complex information, such as prediction and evaluation of multiple outcomes with high relational complexity (Kroger et al., 2002; Vincent et al, 2008). The dorsomedial SFG is also involved in complex cognitive processing, including performance monitoring, action selection and switching in preparation of responses (Ridderinkhof et al., 2004; Rushworth et al., 2004).

One previous study also found structural differences in the white matter of the medial SFG in fighter pilots based on diffusion tensor imaging (Roberts et al., 2010). This structural difference was correlated with cognitive performance, where study participants needed to ignore distracting stimuli that were incongruent with the task they were required to perform. Specifically, fighter pilots in that study showed quicker adaptation to dealing with such incongruencies and this adaptation appears to involve the medial SFG (Roberts et al., 2010). Our results further support the notion that the SFG is a region characteristic to fighter pilots, for which both functional connectivity data as well as structural data of two independent fighter pilot cohort studies support this notion.

### Functional connectivity alterations between vestibular- and visual brain areas

One aim of this study was to investigate whether the alterations in peripheral vestibular signaling in fighter pilots as observed in previous studies (Tribukait et al., 2011; Tribukait and Eiken 2012) could be detected at the level of the brain through neuroimaging analysis. Through a hypothesis-driven analysis, we showed an increase in functional connectivity between the right OP2, a key vestibular cortical area that was chosen as region of interest, and the left visual cortex. Given that sensory conflicts often arise during flights in a fighter jet, fighter pilots are required to cope with this situation and enhance the weight on the sensory input that is most reliable, being visual inspection of the instruments. Alterations in visual- and vestibular functional connectivity have been previously indicated in a group of eleven cosmonauts returning from long-duration space missions. Decreased connectivity of vestibular nuclei, right inferior parietal cortex and cerebellum with motor-, visual-, vestibular and proprioception associated brain areas are thought to contribute to solving mismatching sensory information coming from different sensory systems (Pechenkova et al., 2019). Further evidence from a spaceflight study indicates an increase in visual activity upon vestibular stimulation after spaceflight compared to before, reflecting the reweighting of sensory information (Hupfeld et al., 2022). Finally, a group of patients who suffer from visually induced dizziness exhibit decreased global connectivity in the posterior insular cortex and increased global connectivity in the visual cortex (Van Ombergen et al., 2017a). This provides another example of the dynamic interplay between vestibular and visual cortex when the vestibular system is challenged. Here, we show stronger functional coupling between vestibular and visual cortex in fighter pilots, which may again suggest similar multisensory adaptations of the brain in individuals who are known to be exposed to conflicting sensory information induced by gravity level alterations. This vestibular-visual interaction might be important with respect to proper adaptation to altered gravity levels such as during missions to the Moon and Mars. Specifically, training of space crews could aim at strengthening the coupling between vestibular and visual brain areas to better prepare astronauts for challenging missions.

Next, we investigated whether the right AG, which was known to exhibit reduced global connectivity after gravity level alterations induced by parabolic flight, showed altered functional connectivity in fighter pilots. In the parabolic flight study, the reduced global connectivity in the right AG is associated with its role in the perception of upright and therefore highlights a spatial processing component (Van Ombergen et al., 2017b). In the current study, we find increased functional coupling between right and left AG. One study demonstrated a causal role of the left angular gyrus in left-right discrimination by evaluating such task performance upon transiently inhibiting the left angular gyrus (Hirnstein et al., 2011). Hence, this function appears to complement the spatial processing role of the right AG. Although an exact role of this increased functional coupling requires further clarification, we demonstrate in this study that this specific right AG region exhibits altered functional connectivity in individuals exposed for the first time to gravity alterations induced by parabolic flight, as well as in fighter pilots who experience frequent altered G-levels during flight maneuvers. The AG might therefore play a role in adapting spatial processing strategies induced by gravity level alterations.

### Limitations and future directions

A first limitation of our study concerns the interpretability of our findings with respect to predisposition effects. Our results did not reveal any correlation between flight hours and the connectivity in vestibular cortex, visual cortex or angular gyrus, even though these regions exhibit connectivity differences between pilots and controls. The absence of such findings either points toward a pre-existing neural characteristic in this group of fighter pilots or either to a change that occurs during training prior to flying or after a small number of flight hours. Although the observed differences in functional connectivity between fighter pilots and matched controls could be attributed to increased g-level exposure, our study design does not allow control for predisposition of fighter pilots. These limitations may be addressed by conducting a longitudinal study in fighter pilots. Particularly the observed connectivity differences in brain regions involved in cognitive function could have been present before initiation of the training and therefore reflect a predisposition specific for fighter pilots.

Next, this study is restricted to investigating connectivity differences between fighter pilots and controls in only a few specific brain regions. Other regions involved in proprioception and visual processing were not considered in this study, even though such regions have been shown to have altered activity or connectivity in space crew after spaceflights (Pechenkova et al., 2019; Hupfeld et al., 2022). In this study, we chose to reduce the number of ROIs to avoid accumulation of multiple comparisons and to strongly focus on our main hypotheses. To better understand the central adaptation that is associated with exposure to different gravity levels, future studies could investigate those brain regions associated with various other sensory or sensorimotor functions, such as proprioception.

Furthermore, as we did not include any data on cognitive performance, a direct interpretation of the differences in the functional resting state connectivity between both experimental groups remains suggestive. Increases or decreases in connectivity can both be associated with either beneficial or disadvantageous adaptations. For instance, it was shown in a cohort of 15 astronauts that decreased FC in the right inferior frontal gyrus was associated with improved cognitive performance following long-duration spaceflight, highlighting that decreased connectivity is not necessarily detrimental (Salazar et al., 2022). Hence, FC changes as observed in our study as well as in astronauts could reflect distinct types of adaptation. Therefore, inclusion of behavioral data related to cognitive function, spatial processing, and vestibular function would be beneficial for future neuroimaging studies in fighter pilots to confirm the hypothesized implications of the currently observed functional connectivity characteristics of fighter pilots. Finally, while fighter jet pilots represent an occupational group with extraordinary research interest, there are a limited number of fighter pilots in the Royal Belgian Air Force and access to their data is complicated and subject to several logistic restrictions. As a result, the sample size and statistical power in the present study was relatively low.

## Conclusion

Taken together, these findings suggest that the conditions to which fighter pilots are exposed during their career (increased g-levels, sensory conflicts, and training) alter their brain functional connectivity, reflecting neuroplasticity. This plasticity can be observed as alterations in visual- and vestibular functional coupling, in higher order spatial processing of the brain, and in frontal regions associated with various cognitive functions. These results reveal insights to possible adaptation in individuals exposed to altered gravitational forces and may have implications for spaceflight research. This knowledge can be used to eventually fit training programs for fighter pilots to stimulate the desired connectivity/neuroplasticity, and help making choices between for example training in motion-based flight simulators *versus* non-motion-based flight simulators. Likewise this can be used for optimal training in space crew.

## Data Availability

The original contributions presented in the study are included in the article/supplementary material, further inquiries can be directed to the corresponding author.
